# Quantification of Albumin and γ‑Globulin Concentrations in an Electrolyte Solution by Multivariate-Regressive Gaussian Admittance Relaxation Times Distribution (mgARTD)

**DOI:** 10.1021/acsabm.5c01188

**Published:** 2025-09-08

**Authors:** Arbariyanto Mahmud Wicaksono, Songshi Li, Ryoma Ogawa, Masahiro Takei

**Affiliations:** Department of Mechanical Engineering, Graduate School of Engineering, 12737 Chiba University, Chiba 263-8522, Japan

**Keywords:** Gaussian process, impedance spectroscopy, multivariate regression, protein concentration quantification, relaxation times distribution

## Abstract

Albumin and γ-globulin concentrations in an electrolyte solution have been quantified by a multivariate-regressive Gaussian admittance relaxation times distribution (mgARTD). The mgARTD is built based on the training data consisting of the impedance spectroscopy system measurement result of protein mixture solutions with a known concentration of albumin, γ-globulin, and sodium electrolyte to perform concentration quantification on a prospective protein mixture solution with an unknown concentration. The mgARTD consists of three steps: (1) Prediction step of the peak matrix P* by Gaussian ARTD (gARTD) with the Gaussian process and peak detection algorithm, (2) Training step of the approximated coefficient matrix **Ã** based on the multivariate-regressive formula P* = **Ac** + **φ** (**A**: multivariate-regression coefficient matrix, **φ**: error matrix, and **c**: known concentration matrix of the training data set), and (3) Quantification step of the approximated concentration **c̃** based on the Gauss–Newton algorithm from the predicted $\overset{ˇ}{P^{*}}$ of the quantification data and the approximated **Ã**. In the experiments, first, **Ã** is approximated on the predicted P* and the known **c** under the 27 cases of protein and electrolyte concentrations (albumin: 0.800–2.400 g/dL, γ-globulin: 0.400–1.200 g/dL, and sodium electrolyte: 0.700–0.750 g/dL). Next, **c̃** is determined by **Ã** and quantification data $\overset{ˇ}{P^{*}}$ under an unknown albumin, γ-globulin, and sodium electrolyte concentration. The effect of albumin and γ-globulin on ARTD is observed in $\hat{\tau_{1}^{*}\;}$ > 0.05 ms for counterion effects and in 10 μs > $\hat{\tau_{2}^{*}\;}$ > 1 μs for protein reorientations. The gARTD γ* function achieves a transform fitting error of 10.90% on average, which is 12.12% lower than that of ARTD and minimizes the quantification error. The absolute percentage quantification error of **c̃** by mgARTD is 9.90%, 9.12%, and 1.70% for albumin, globulin, and sodium electrolyte, respectively, which is significantly lower than 25.56%, 20.90%, and 25.71% for albumin, globulin, and sodium electrolyte by mARTD.

## Graphical abstract

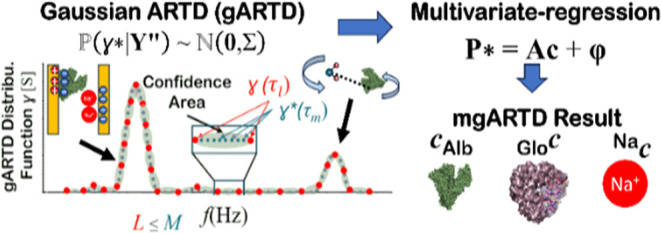

## 1 Introduction

The quantification of albumin^1^ and γ-globulin concentration in electrolyte interstitial fluids is crucial in clinical chemistry because these proteins maintain osmotic balance and immune response.^2^ Although albumin and γ-globulin are typically quantified from the sample of blood or urine by ELISA,^3^ on-site monitoring from the human body is highly demanded instead of the sample-based measurement because the changes in interstitial fluids occur at hourly rates in the body and conventional method are time-consuming.^4^ Recently, the application of electrical impedance spectroscopy (EIS) to clinical chemistry has shown a great on-site monitoring potential^5,6^ for quantification of protein concentration in an electrolyte interstitial fluid.^7,8^ EIS injects a multifrequency alternating current to induce relaxation phenomena between the protein electrical charge and the surrounding electrolytes.^9^ EIS enables an inexpensive reagent-less quantification which reduces the measurements operation cost.^10^ Reagents used in conventional ELISA bind each quantified substance with high accuracy, which increases the operation cost of multiple substance concentration quantification. A reagentless spectrophotometry device, such as a NanoDrop device, is only able to quantify the total protein concentration or a single substance target with an expensive equipment cost. An understanding of the influence of protein and nonprotein substances on electrical spectroscopy is essential to achieve quantification of protein concentration.^11^ Figure 1 shows the relaxation phenomena representing the interaction of alternating current fields in EIS. Scientifically, a low-frequency current causes a counterion to form at high relaxation times (*τ* ≤ 10 ms)^12^ based on the total charged molecules in the protein and electrolyte. A higher frequency current causes the protein β dispersion from the protein–water reaction at low relaxation times (10 μs ≤ *τ* ≤ 1 μs).^13^ Nevertheless, the quantification of albumin and γ-globulin by EIS is not easy technologically because the effect of the electrolyte counterion and the electrode double layer^14^ obscures the EIS interpretation.

**Figure 1:**
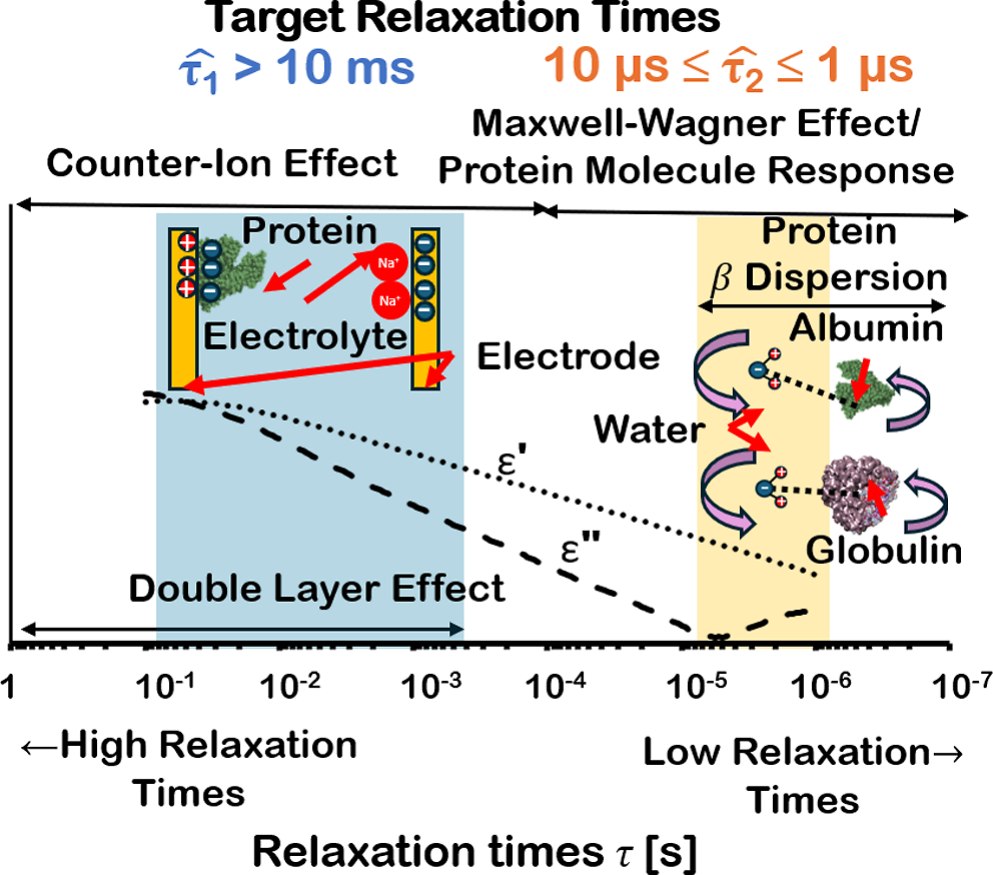
Target relaxation times phenomena in EIS measurement for quantification of protein concentration.

In order to easily understand EIS interpretation, impedance-based relaxation times distribution (RTD) predicts the relaxation times *τ* to highlight the relaxation phenomena into a narrower time-domain range in the form of frequency-dependent relaxation times-domain function *γ*.^15^ Table 2 shows the progression of the RTD for concentration quantification. RTD is a model-free analytical approach and does not require specific prior knowledge about the measured substances by transforming frequency-domain measurement result into *γ* function.^16^ Despite the improvement provided by RTD, three main issues limit the application of impedance-based RTD for concentration quantification of albumin and γ-globulin in an electrolyte solution, which are (1) the counterion effect is overlapping at high relaxation times between the electrolyte and protein,^17^ as shown in Figure 1, which causes inaccuracies in the quantification of protein concentration, (2) protein β dispersion of albumin and γ-globulin is overlapping at low relaxation times,^18^ as shown in Figure 1, which causes impedance-based RTD unable to quantify the albumin and γ-globulin concentration separately, and (3) the ill-posed problem of RTD transform requires a complex calculation to solve RTD and minimize the transform fitting error.^19^

**Table 1:** Table of Nomenclature

symbol	description
○*	predicted variable
○̃	approximated variable
○̌	quantification data variable
○^**+**^	training data variable
P* $\overset{ˇ}{P^{*}}$	predicted peak matrix of the training data set predicted peak matrix of the quantification data
**A**	multivariate-regression coefficient
**Ã**	approximated multivariate-regression coefficient
**c**	known concentration of the training data set
**c̃**	approximated concentration of the quantification data
**φ**	error matrix
**Y** ^+^	measured admittance of the training data set
**Y̌**	measured admittance of the quantification data
**○**″	imaginary component of the variable
**Y** ^+^″	measured imaginary admittance of the training data set
γ*	predicted gARTD function
$\hat{\gamma^{*}\;}$	predicted peak points of the gARTD function
$\hat{\tau^{*}\;}$	predicted relaxation times of the gARTD function
	

**Table 2:** Development of Admittance Relaxation Times Distribution (ARTD) for Concentration Quantification

methodology		substance	reference
impedance-based analysis	RTD	electrolyte in a battery	TH. Wan et al.^16^
admittance-based analysis	ARTD	protein in a solution and adipose tissue	A.M. Wicaksono et al.^21^
	mARTD	protein in adipose tissue	A.M. Wicaksono et al.^20^
	mgARTD	albumin and γ-globulin in a solution	this research

In order to achieve a separate quantification of albumin and γ-globulin in an electrolyte solution, we previously proposed multivariate-regressive ARTD (mARTD) to quantify protein concentration^20^ by forming a statistical relationship between admittance RTD (ARTD) analysis results to protein concentration and separating the electrolyte concentration impact. We have previously proposed ARTD to highlight the protein concentration^21^ impact on EIS measurement results, which shows ARTD results has a higher linear correlation to protein concentration in comparison to conventional RTD. The ARTD is an excellent tool for separating the impact of protein concentration occurring on lower relaxation times from the more impactful effect of the electrolyte counterion and electrode double layer occurring at high relaxation times.^22^ Furthermore, ARTD shows a linear correlation between protein concentration β dispersion to the lower relaxation times of the ARTD result, which is not present in RTD. The presence of the two linear correlations of low and high relaxation times in ARTD enables the separate quantification of albumin and γ-globulin concentration by mARTD regardless of the fluctuation of sodium electrolyte concentration. As previously noted, the lack of this two-relaxation-time phenomenon in RTD further prevents the integration of multivariate regression into RTD for quantifying albumin and γ-globulin concentrations. Despite the separation and quantification ability of mARTD to quantify albumin and γ-globulin concentration, the ill-posed problem of RTD transform causes inaccuracies in mARTD quantification, resulting in a high quantification error.^20^

Therefore, in this study, the authors propose an idea of a multivariate-regressive Gaussian admittance relaxation times distribution (mgARTD) to quantify albumin and γ-globulin separately with high accuracy. The Gaussian process impedance-based RTD (gRTD) provides probabilistic modeling and the Bayesian interface to solve RTD. The gRTD provides a lower transform fitting error in comparison to impedance-based RTD.^23^ The application of the Gaussian process shows promising potential for the concentration quantification of electrolytes in battery^24^ and biological tissue analysis.^25^ The integration of gRTD to mARTD formed mgARTD, which provides a better quantitative correlation between ARTD to albumin and the γ-globulin concentration. The proposed mgARTD shows a great potential in multiple substance concentration quantification with a low cost and reagentless method as well as greater accuracy to mARTD. The application of mgARTD has three significant possibilities: namely, (1) quantification of protein concentration under a fluctuating background electrolyte by ARTD, (2) albumin and γ-globulin concentration separation by a multivariate-regressive model, and (3) minimization of ARTD transform error and quantification error by the Gaussian process.

This study aims to achieve three main objectives, which are (1) to observe the effect of albumin, γ-globulin, and electrolyte concentration on gARTD, (2) to assess the reduction in transform fitting error by the gARTD, and (3) to evaluate the albumin and γ-globulin concentration quantification accuracy of mgARTD in comparison to mARTD.

## 2 Multivariate-Regressive Gaussian Admittance Relaxation Times Distribution (MGARTD)

### 2.1 Overview of ARTD^21^

Figure 2 shows the extraction of *γ* function in ARTD based on the modified Voigt-Maxwell circuit,^26^ which is part of the proposed mgARTD (see Table 1 for the Table of Nomenclature). The modified Voigt-Maxwell circuit consists of conductance of electrode surface *G*_∞_, principle admittance of charge transfer *G*_0_, principle capacitance of charge transfer *C*_0_, and *M* number of Maxwell circuits arranged in parallel to approximate the RTD (1 ≤ *m* ≤ *M*).^27^ The relaxation times *τ*_*m*_ of each Maxwell circuit are calculated by *τ*_*m*_ = *C*_*m*_/*G*_*m*_. The high *τ*_*m*_ relates to the relaxation times of counterions,^12^ and the lower *τ*_*m*_ relates to the relaxation times of protein β dispersion.^13^ The relationship between the measured admittance *Y*_*l*_ at the *l*th injected current frequency *f*_*l*_ (1 ≤ *l* ≤ *L*) and γ function of logarithmic relaxation times ln *τ*_*l*_ is expressed by

$$Y_{l}=Y_{l}^{\prime }+jY_{l}^{\prime \prime },Y_{l}∼\tilde{Y_{l}}$$

$$\overset{∼}{Y_{l}}=\frac{1}{\frac{1}{G_{\infty }}+\frac{1}{G_{0}+j\tau_{l}\left(C_{0}+\int_{0}^{\infty }\frac{\gamma \left(\ln{\;\tau }_{m}\right)}{1+j\tau_{l}\tau_{m}}\;d\tau_{m}\right)}}$$ 1

where *Y*_*l*_′ is the real admittance component, *Y*_*l*_″ is the imaginary admittance component, *j* is the imaginary number, $\tilde{Y_{l}}$ is the approximated admittance by ARTD, and *τ*_*l*_ is the relaxation time of the injected current frequency (*τ*_*l*_ = (2π*f*_*l*_)^−1^). The extraction of γ from *Y*_*l*_ is an ill-posed problem if *L* < *M*,^19^ which means γ function inversion from *Y*_*l*_ is not simple, and the solution is sensitive to experimental errors. Conventionally, *γ* is expressed by a linear weight series *x*_*m*_ and a normal distribution function *λ*_*m*_ to extract the *τ*_*m*_ distribution from the measured imaginary admittance *Y*″.^21^ However, the actual measurement relaxation phenomenon does not always follow the linear series of the normal distribution function. This study proposes Gaussian process to predict γ* from the measured *Y*″, which is more versatile in determining *τ*_*m*_ distribution.^24^

**Figure 2:**
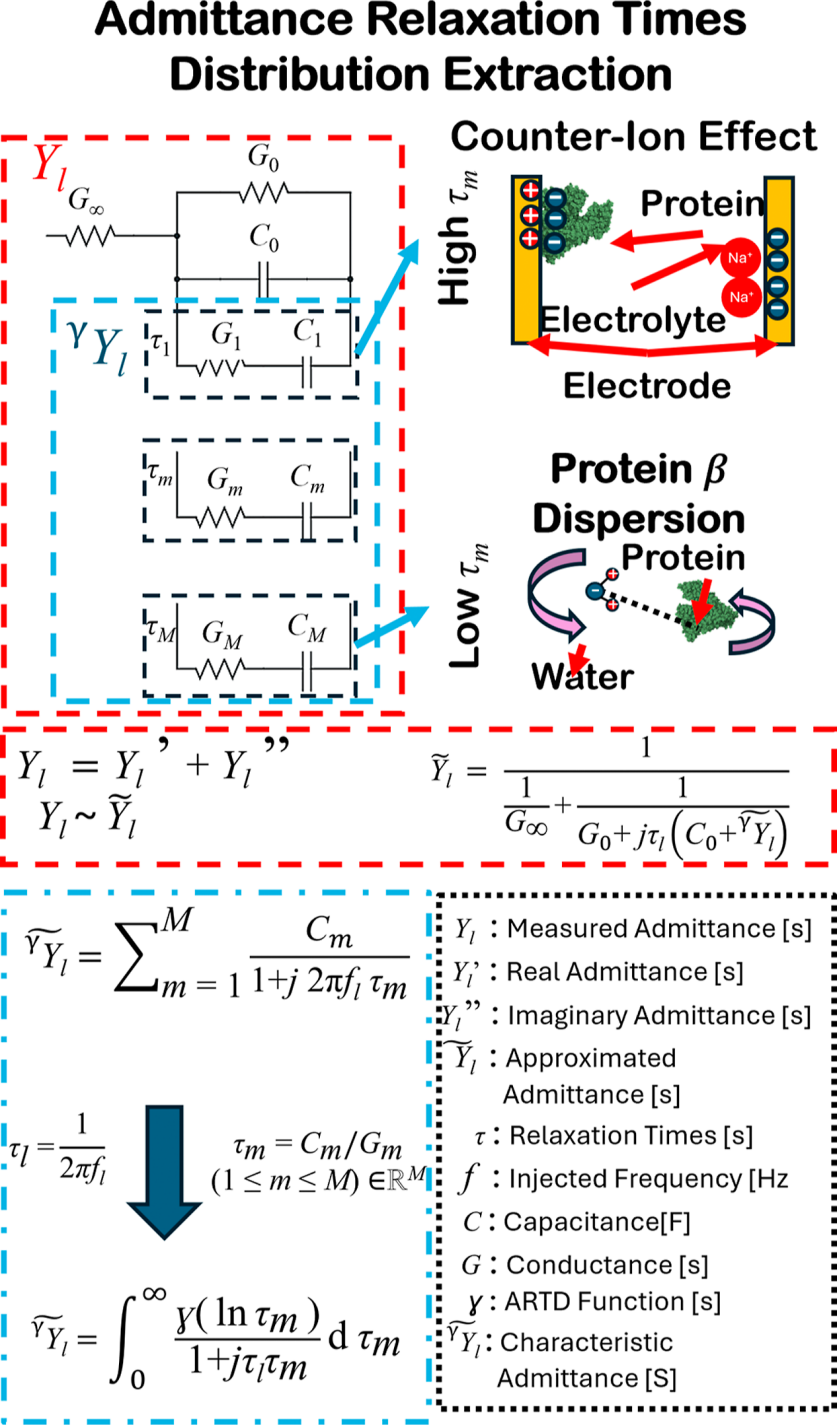
ARTD formulation.

### 2.2 Overview of mgARTD

Figure 3 (see Table 1 for the Table of Nomenclature) shows the conceptual figure of multivariate-regressive Gaussian admittance relaxation times distribution (mgARTD) which consists of (1) Prediction step of the peak matrix P*, (2) Training step of the coefficient matrix **Ã**, and (3) Quantification step of the approximated concentration matrix **c̃**.

**Figure 3:**
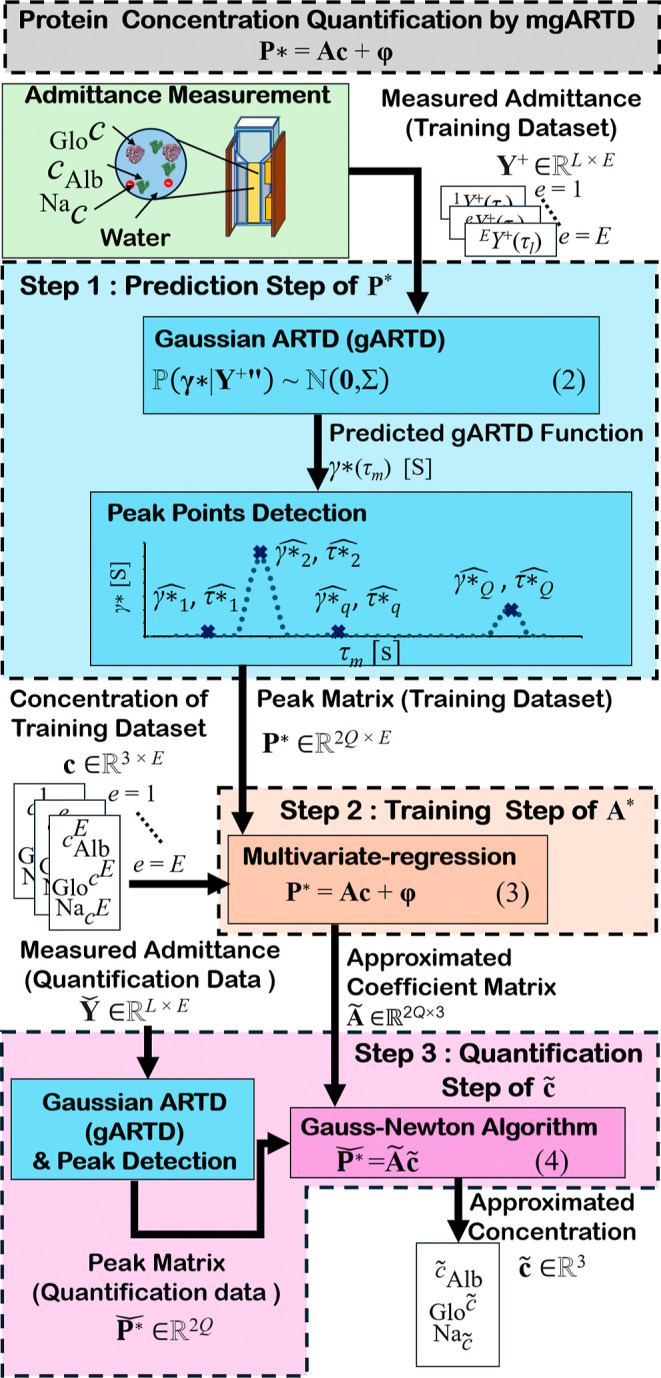
Quantification of protein concentration conceptual figure based on multivariate-regressive Gaussian admittance relaxation times distribution (mgARTD).

In the first Prediction step, the peak matrix P* ∈ R ^2*Q*×*E*^ (*q*: number of peak points in the γ function (1 ≤ *q* ≤ *Q*), *E*: number of training data set (1 ≤ *e* ≤ *E*)) is the training data set, which consists of peak points $\hat{\gamma^{*}\;}$ of the predicted γ* function and the corresponding relaxation times $\hat{\tau^{*}\;}$ . The γ* function is predicted from the measured imaginary admittance **Y″** = [*Y*_1_″, ..., *Y*_*l*_″, ..., *Y*_*L*_″]^T^ ∈ R ^*L*^ by Gaussian ARTD (gARTD)^21^ following

2 $$P\left(\gamma *|Y^{\prime \prime }\right)∼N\left(0,\Sigma \right)$$

where P is the posterior distribution, N is the zero mean Gaussian distribution, and **Σ** is the covariance matrix. The peak points detection algorithm acquires the $\hat{\gamma^{*}\;}$ and the $\hat{\tau^{*}\;}$ from γ*­(ln *τ*_*m*_) to predict P* as the basis for the correlation between relaxation phenomena and substance concentrations.

In the second Training step, mgARTD forms a regression model to achieve the concentration quantification of three substances: albumin *c*_Alb_, γ-globulin _Glo_ *c*, and sodium electrolyte ^Na^ *c*. The regression model in this study is represented by the coefficient matrix **A** ∈ R ^2*Q*×3^ which follows

3 $$P^{*}=Ac+\varphi$$

where **c** ∈ R ^3×*E*^ is the known matrix of substance (*c*_Alb_, _Glo_ *c*, and ^Na^ *c*) concentrations and **φ** ∈ R ^2*Q*^^×^^*E*^ is the defined error matrix from a three-dimensional Gaussian distribution N _3_ following **φ** ∼ N_3_(0, Σ). The measured admittance of training data set **Y** ^+^ ∈ R ^*L*×*E*^ forms the approximated **Ã** from the regression model training to enable the concentration quantifications.

In the third Quantification step, the approximated **c̃** ∈ R ^3^ is calculated by the Gauss–Newton algorithm as follows:

4 $$\overset{ˇ}{P^{*}}=Ã\mathrm{c̃}$$

where $\overset{ˇ}{P^{*}}$ ∈ R ^2*Q*^ is the predicted peak matrix from the measured admittance of quantification data **Y̌** ∈ R ^*L*^.

### 2.3 Step 1: Prediction Step of the Peak Matrix P

The gARTD predicts the *γ* function through probabilistic modeling and the Bayesian interface. The predicted RTD function γ* = [*γ*_1_*, ..., *γ*_m_*, ..., γ*_M_]^T^ ∈ R ^*M*^ ^21^ in gARTD is predicted from **Y**″ based on eq 2 following

$$\left(\begin{array}{l} Y^{\prime \prime }\\ \gamma^{*} \end{array}\right)=\left(\begin{array}{l} \begin{array}{l} \begin{array}{l} Y_{l}^{\prime \prime }\\ \vdots \end{array}\\ \begin{array}{l} Y_{l}^{\prime \prime }\\ \vdots \end{array}\\ Y_{l}^{\prime \prime } \end{array}\\ \begin{array}{l} \begin{array}{l} \gamma_{l}^{*}\\ \vdots \end{array}\\ \begin{array}{l} \gamma_{m}^{*}\\ \vdots \end{array}\\ \gamma_{M}^{*} \end{array} \end{array}\right)∼N\left(0,\left(\begin{array}{ll} L^{2}K+\theta_{1}^{2}I & LK^{*}\\ LK^{{*}^{T}} & K^{**} \end{array}\right)\right)$$ 5

where *θ*_1_ is the noise level, **I** ∈ R ^*L*×*L*^ is an identity matrix, and L(.) is the linear transform function. The covariance matrices **K** ∈ R ^*L*×*L*^, K* ∈ R ^*L*×*M*^, and K** ∈ R ^*M*×*M*^ are defined by the kernel function *k* ^21^ expressed by

$$K=\left[\begin{array}{l} k\left(\ln\tau_{l,}\ln\tau_{l}\right)\\ \begin{array}{l} \vdots \\ k\left(\ln\tau_{l,}\ln\tau_{l}\right)\\ \vdots \end{array}\\ k\left(\ln\tau_{L,}\ln\tau_{l}\right) \end{array}\begin{array}{l} ...\\ \begin{array}{l} \ddots \\ ...\\ \ddots \end{array}\\ ... \end{array}\begin{array}{l} k\left(\ln\tau_{l,}\ln\tau_{l}\right)\\ \begin{array}{l} \vdots \\ k\left(\ln\tau_{l,}\ln\tau_{l}\right)\\ \vdots \end{array}\\ k\left(\ln\tau_{L,}\ln\tau_{l}\right) \end{array}\begin{array}{l} ...\\ \begin{array}{l} \ddots \\ ...\\ \ddots \end{array}\\ ... \end{array}\begin{array}{l} k\left(\ln\tau_{l,}\ln\tau_{L}\right)\\ \begin{array}{l} \vdots \\ k\left(\ln\tau_{l,}\ln\tau_{L}\right)\\ \vdots \end{array}\\ k\left(\ln\tau_{L,}\ln\tau_{L}\right) \end{array}\right]$$ 6

7 $$K^{*}=\left[\begin{array}{l} k\left(\ln\tau_{1,}\ln\tau_{1}^{*}\right)\\ \begin{array}{l} \vdots \\ k\left(\ln\tau_{l,}\ln\tau_{1}^{*}\right)\\ \vdots \end{array}\\ k\left(\ln\tau_{L,}\ln\tau_{1}^{*}\right) \end{array}\begin{array}{l} ...\\ \begin{array}{l} \ddots \\ ...\\ \ddots \end{array}\\ ... \end{array}\begin{array}{l} k\left(\ln\tau_{l,}\ln\tau_{m}^{*}\right)\\ \begin{array}{l} \vdots \\ k\left(\ln\tau_{l,}\ln\tau_{m}^{*}\right)\\ \vdots \end{array}\\ k\left(\ln\tau_{L,}\ln\tau_{m}^{*}\right) \end{array}\begin{array}{l} ...\\ \begin{array}{l} \ddots \\ ...\\ \ddots \end{array}\\ ... \end{array}\begin{array}{l} k\left(\ln\tau_{1,}\ln\tau_{M}^{*}\right)\\ \begin{array}{l} \vdots \\ k\left(\ln\tau_{l,}\ln\tau_{M}^{*}\right)\\ \vdots \end{array}\\ k\left(\ln\tau_{L,}\ln\tau_{M}^{*}\right) \end{array}\right]$$

8 $$K^{**}=\left[\begin{array}{l} k\left(\ln\tau_{1,}^{*}\ln\tau_{1}^{*}\right)\\ \begin{array}{l} \vdots \\ 0\\ \vdots \end{array}\\ 0 \end{array}\begin{array}{l} ...\\ \begin{array}{l} \ddots \\ ...\\ \ddots \end{array}\\ ... \end{array}\begin{array}{l} 0\\ \begin{array}{l} \vdots \\ k\left(\ln\tau_{m,}^{*}\ln\tau_{m}^{*}\right)\\ \vdots \end{array}\\ 0 \end{array}\begin{array}{l} ...\\ \begin{array}{l} \ddots \\ ...\\ \ddots \end{array}\\ ... \end{array}\begin{array}{l} 0\\ \begin{array}{l} \vdots \\ 0\\ \vdots \end{array}\\ k\left(\ln\tau_{M,}^{*}\ln\tau_{M}^{*}\right) \end{array}\right]$$

where the covariance matrices comprised of all the τ pairs between ln *τ_l_* and ln *τ_m_* * from the matrices **τ** = [*τ*_1_, ..., *τ_l_*, ..., *τ_L_*]^T^ ∈ R ^*L*^ and τ* = [*τ*_1_*, ..., *τ_m_* *, ..., *τ_M_* *]^T^ ∈ R ^*M*^. The kernel function *k* (ln *τ_l_*_,_ *τ_m_* *) is formulated as a Gaussian kernel by

$$k\left(\ln\tau_{l,}\ln\tau_{m}^{*}\right)=\theta_{2}^{2}\exp\left(‐\frac{{\left|\ln\;\tau_{l}-\ln\tau_{m}^{*}\right|}^{2}}{2\theta_{3}^{2}}\right)$$ 9

where *θ*_2_ is the vertical variation parameter and *θ*_3_ is the scale length. The optimal hyperparameter **θ** = [*θ*_1_, *θ*_2_, *θ*_3_]^T^ in eqs 5 and 9 is determined by maximizing the log marginal likelihood formulated by

$$\log p\left(Y^{\prime \prime }|\ln\tau ,\theta \right)=-\frac{1}{2}Y^{T}{\left(L^{2}K+\theta_{1}^{2}I\right)}^{-1}Y^{\prime \prime }$$

10 $$-\frac{1}{2}\log|\left(L^{2}K+\theta_{1}^{2}I\right)|-\frac{N}{2}\log\left(2\pi \right)$$

where **Y**″ is acquired from **Y** = [*Y*_1_, ..., *Y*_*l*_, ..., *Y*_*L*_]^T^ ∈ R ^*L*^, which is composed of the complex admittance *Y*_*l*_ = *Y*_*l*_′ + *Y*_*l*_″ (*Y*_*l*_′: real component of admittance).

Plotting the resolved γ*­(ln *τ_m_*) from eq 5 produces the relaxation time distribution plot with multiple peaks, which highlight the substance-specific relaxation phenomena. The peak points $\hat{\gamma^{*}\;}$ and $\hat{\tau^{*}\;}$ are predicted by a peak points detection algorithm from the predicted γ* function of the training data set **Y** ^+^ and formulate the predicted P* following

11 $$P^{*}=\left[\begin{array}{l} \begin{array}{l} \hat{\gamma_{1}^{*1}\;}...\hat{\gamma_{1}^{*e}\;}...\hat{\gamma_{1}^{*E}\;}\\ \vdots \ddots \vdots \ddots \vdots \\ \begin{array}{l} \hat{\gamma_{q}^{*1}\;}...\hat{\gamma_{q}^{*e}\;}...\hat{\gamma_{q}^{*E}\;}\\ \vdots \ddots \vdots \ddots \vdots \\ \hat{\gamma_{Q}^{*1}\;}...\hat{\gamma_{Q}^{*e}\;}...\hat{\gamma_{Q}^{*E}\;} \end{array} \end{array}\\ \begin{array}{l} \hat{\tau_{1}^{*1}\;}...\hat{\tau_{1}^{*e}\;}...\hat{\tau_{1}^{*E}\;}\\ \vdots \ddots \vdots \ddots \vdots \\ \begin{array}{l} \hat{\tau_{q}^{*1}\;}...\hat{\tau_{q}^{*e}\;}...\hat{\tau_{q}^{*E}\;}\\ \vdots \ddots \vdots \ddots \vdots \\ \hat{\tau_{Q}^{*1}\;}...\hat{\tau_{Q}^{*e}\;}...\hat{\tau_{Q}^{*E}\;} \end{array} \end{array} \end{array}\right]$$

where $\hat{\gamma_{q}^{*e}\;}$ is the γ* function *q*th peak point acquired from the *e*th training data set and $\hat{\tau_{q}^{*e}\;}$ is the corresponding τ of the peak point.

### 2.4 Step 2: Training Step of Approximated *Ã* ^20^

The Training step of mgARTD requires the known **c** and the predicted P* from **Y** ^+^ to train the undefined **A** following eq 3. The covariance-weighted least-square method defines the approximated **Ã** matrix following

12 $$Ã={\left(c^{T}{\left(I_{2Q}⊗\alpha_{0}\right)}^{‐1}c\right)}^{-1}c^{T}{\left(I_{2Q}⊗\alpha_{0}\right)}^{-1}P^{*}$$

where **α**_**0**_ ∈ R ^2*Q*×2*Q*^ is the defined covariance matrix and **I**_2*Q*_ ∈ R ^2*Q*×2*Q*^ is an identity matrix. The known substance concentration in the training data set is contained in the matrix **c** which is written as follows:

13 c=[cAlb1...cAlbe...cAlbEc1Glo...ceGlo...cEGloc1Na...ceNa...cENa]

where ***c*** ^***e***^ is the known substance concentration of the ***e*** th training data set.

### 2.5 Step 3: Quantification Step of Approximated *c̃*

The **Ã** from the Training step enables the substance concentration quantification by fitting the predicted matrix $\overset{ˇ}{P^{*}}$ ∈ R^2^ ^*Q*^ from the measured admittance of quantification data **Y̌**. Modifying eq 4 enables quantification with **Ã** as a constant variable of the multiple linear regression model and $\overset{ˇ}{P^{*}}$ as the independent variable to quantify the unknown approximated concentration matrix **c̃** with the equation

14 Ã[c̃Albc̃Gloc̃Na]=[γ1*ˇ⋮γq*ˇ⋮γQ*ˇτ1*ˇ⋮τq*ˇ⋮τQ*ˇ]

where $\overset{ˇ}{\gamma_{q}^{*}}$ is the γ* function *q*th peak point and $\overset{ˇ}{\tau_{q}^{*}}$ is the peak point corresponding *τ*. The $\overset{ˇ}{P^{*}}$ from **Y̌** and the **c̃** of the quantification data are not included in the training data set in eq 12. The forward solution to quantify **c̃** follows the Gauss–Newton algorithm by minimizing the residual ς following

15 $$\varsigma \underset{\mathrm{c̃}\in R}{=\arg\;\min}\overset{ˇ}{P^{*}}-Ã\mathrm{c̃}$$

The Gauss–Newton iterative method was applied to obtain the minimization with the formula

16 $${\mathrm{c̃}}^{o+1}={\mathrm{c̃}}^{o}-\left({Ã}^{T}Ã+\psi Ι\right){\left({Ã}^{T}\varsigma \right)}^{-1}$$

where *ψ* is the Gauss hyperparameter, Ι ∈ R ^3×3^ is an identity matrix, and *o* is the iteration number of the minimization. An L-curve method was applied to determine an optimized value of *ψ*.^21^

## 3 Experimental Section

### 3.1 Experimental Setup

Figure 4 shows the experimental setup which comprises an impedance analyzer (IM3536, Hioki EE Corporation Japan), coaxial cable connectors, parallel electrode pairs, printed circuit board (PCB) brackets, an adjustable vice, a cuvette, and a personal computer. The analyzer controlled by the personal computer measures admittance spectra. The four coaxial cables connect the impedance analyzer to the pair of 25 mm × 15 mm parallel electrodes on a PCB. The PCB is glued to an adjustable vice by a PCB bracket. A cuvette with a 0.4 mm gap and a 20 mm × 12 mm contact area is placed between the electrode pair with a conductive paste interface, as shown in Figure 4. A two-load short- and open-automatic calibration was done prior to the measurement to ensure measurement accuracy. Admittance measurement with an injection current frequency of *f* = 10 Hz to 1 MHz is distributed logarithmically with *l* = 201 data points at 0.1 mA. An average value is obtained by automatically repeating each measurement three times.

**Figure 4:**
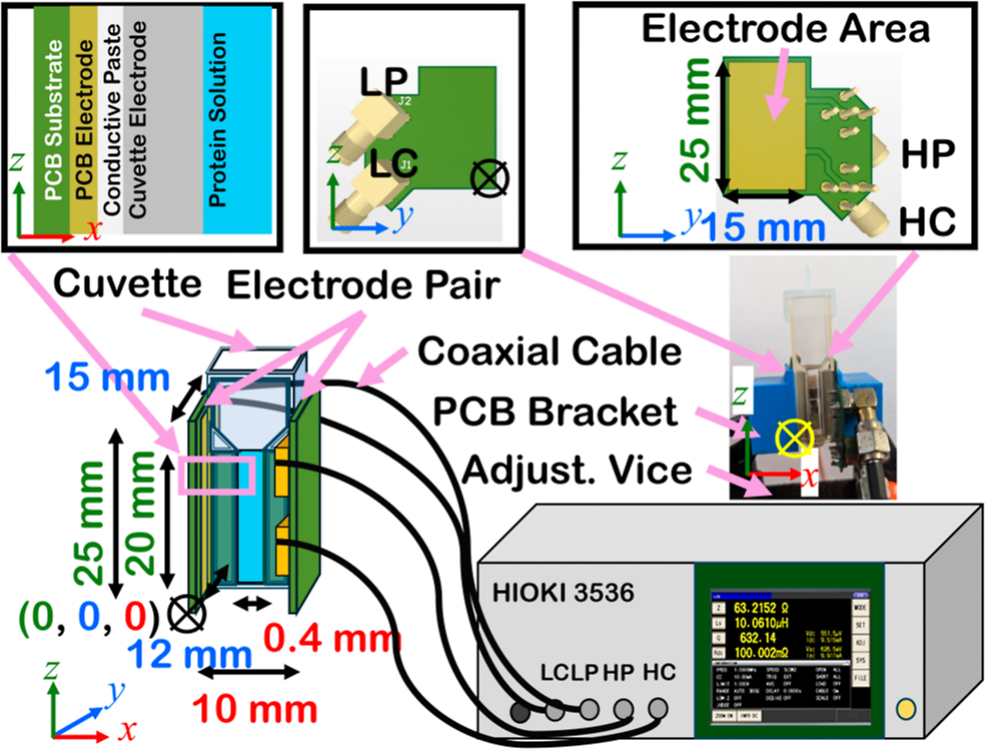
Protein solution measurement setup.

### 3.2 Experimental Condition

The protein solution comprises NaCl salt (Lot No. J190619001) to represent sodium electrolyte, bovine γ-globulin (Lot No. QR15242), and bovine serum albumin (Lot No. LEE0162). Figure 5 shows the twenty-seven training cases (*E* = 27) consisting of *c*_Alb_ = 0.800 to 2.400 g/dL, _Glo_ *c* = 0.400 to 1.200 g/dL, and ^Na^ *c* = 0.700 to 0.750 g/dL combinations. The albumin concentration is based on the normal interstitial fluid value of 1.248 g/dL.^21,28^ The *c*_Alb_, _Glo_ *c*, and ^Na^ *c* are mixed into a 1.2 mL protein solution inside the cuvette.

**Figure 5:**
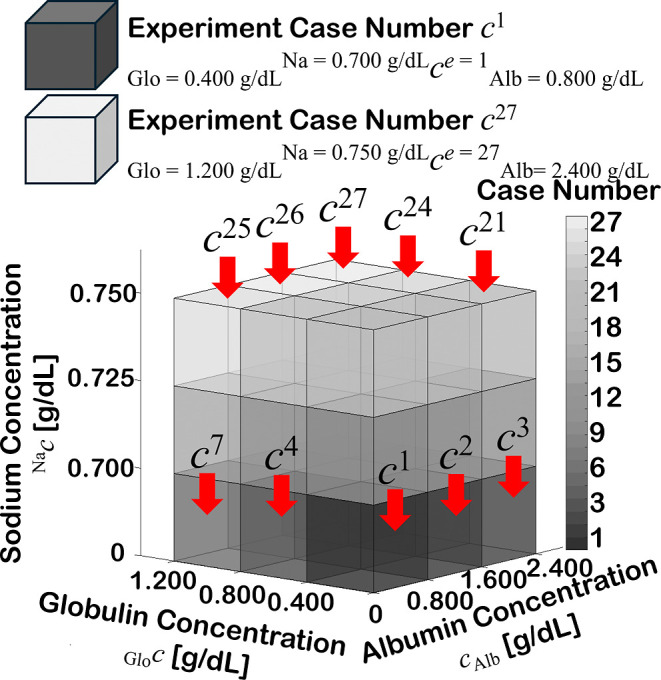
3D plot representation of the twenty-seven training data set. Each case in the data set is represented by _Glo_^Na^ *c* ^*e*^_Alb_ (*e* = training case number).

### 3.3 Experimental Method

The γ* function of gARTD in the Prediction step is compared to the conventional *γ* function of ARTD to assess the fitting accuracy. The P* in both methods is acquired by a peak point detection algorithm. The **Ã** in the Training step is approximated based on the predicted P* and the known **c** under the 27 cases of protein and electrolyte concentrations (albumin: 0.800–2.400 g/dL, globulin: 0.400–1.200 g/dL, and sodium electrolyte: 0.700–0.750 g/dL). The unknown approximated concentration **c̃** is determined by **Ã** and the predicted **P̌** from admittance measurement under similar measurement conditions to the training data set in the Quantification step. The quantification accuracy of mgARTD is compared to mARTD in order to see the improvement in quantification accuracy. In terms of peak points detection of P* in the first Prediction step, $\hat{\gamma^{*}\;}$ = [ $\hat{\gamma_{1}^{*}\;}$ ,..., $\hat{\gamma_{q}^{*}\;}$ ,···, $\hat{\gamma_{Q}^{*}\;}$ ]^T^ ∈ R ^*Q*^ and $\hat{\tau^{*}\;}$ = [ $\hat{\tau_{1}^{*}\;}$ ,···, $\hat{\tau_{q}^{*}\;}$ ,···, $\hat{\tau_{Q}^{*}\;}$ ]^T^ ∈ R ^*Q*^ are detected by the algorithm

dγ*(ln⁡τm)dτ=0dγ*(ln⁡τm+1)dτ≤0dγ*(ln⁡τm−1)dτ>0γ*(ln⁡τm)≥γlim}τm∈Rm,γ*^=[γ*(ln⁡τm)],τ*^=[τm] 17

where *γ*_lim_ is determined to eliminate local maxima under a set value. The matrixes γ̂ and **τ̂** contain *Q* number of peaks acquired from the peak points detection algorithm. The peak points detection of $\overset{ˇ}{P^{*}}$ in the third Quantification step follow the same algorithm to detect ***γ* ˇ** = [ $\overset{ˇ}{\gamma_{1}}$ ,..., $\overset{ˇ}{\gamma_{q}}$ ,···, γQ^ ]^T^ ∈ R ^*Q*^ and **τ̌** = [ $\overset{ˇ}{\tau_{1}}$ ,..., $\overset{ˇ}{\tau_{q}}$ ,..., $\overset{ˇ}{\tau_{Q}}$ ]^T^ ∈ R ^*Q*^.

In terms of assessing the reduction in transform fitting error and quantification accuracy of gARTD in the Prediction step, extraction of the γ function by conventional ARTD is conducted as a comparative method. In the case of ARTD, the *γ* function is extracted from the normal distribution function^21^ of the Maxwell circuits with a linear weight series. The normal distribution function of the *m*th Maxwell circuit *λ_m_* is written as

$$\lambda_{m}=\exp\left({\left(-\mu \ln\tau_{m}\right)}^{2}\right)$$ 18

where the exp variable represents the Napier constant and μ is the shape factor. The extracted *γ* function in the conventional ARTD is expressed by

$$\gamma \left(\ln\tau \right)=\overset{M}{\underset{m=1}{\sum }}x_{m}\lambda_{m}\left(\ln\tau \right)$$ 19

with the *m*th linear weight *x*_*m*_ to adjust the *λ_m_* function amplitude to fit to **Y″**. The ridge regression algorithm to calculate imaginary admittance approximation **Ỹ″** following

$$\underset{x_{m}\in R}{x=\arg\;\min}{∥Y^{\prime \prime }-{Ỹ}^{\prime \prime }∥}_{2}^{2}+{\delta ∥hx∥}_{2}^{2}$$ 20

where *δ* is the regularization parameter for the differential matrix **h** ∈ R ^*L*×*M*^. The extracted peak matrix **P** ∈ R ^2*Q*×*E*^ is composed of the extracted γ function peak points. In terms of the mgARTD and mARTD in the Training step, **Ã** is approximated to quantify *c*_Alb_, _Glo_ *c*, and sodium electrolyte ^Na^ *c*. The known substance concentration is contained in matrix **c** ∈ R ^3×27^. Equation 12 approximates **Ã** in the Training step with the known **c** of the training data set as well as the P* matrix for mgARTD and the **P** matrix for mARTD.^20^

In terms of the mgARTD and ARTD quantification accuracy in the Quantification step, the **Ã** acquired from the Training step quantifies *c̃*_Alb_, _Glo_ *c̃*, and ^Na^ *c̃* in the unknown **c̃** matrix by fitting the predicted matrix $\overset{ˇ}{P^{*}}$ from the quantification data into eq 14. The quantified **c̃** accuracy is assessed by calculating the absolute percentage quantification error ϵ with the formula

21 $$\epsilon =\left|\frac{c-\mathrm{c̃}}{c}\right|\times 100\%$$

where *c* is the known analyte concentration and *c̃* is the approximated concentration by mgARTD and mARTD.

## 4 Experimental Result

### 4.1 Prediction of P* from gARTD and ARTD

The gARTD and ARTD transform of the admittance measurement provides the γ*­(ln *τ*_*m*_) plot and *γ* (ln *τ*_*m*_) plot, respectively, to show the impact of protein and sodium electrolyte concentration on the RTD. Calculation of gARTD and ARTD is based on the fitting of γ* and *γ* function to approximate ${Ỹ}^{\prime \prime }$ , as shown in Equations 2 and 22. Figure 6a shows the imaginary admittance fitting result of gARTD and ARTD. The result demonstrates that gARTD provides a better transform fitting error result in comparison to ARTD in the case *c* ^9^. Results from gARTD and ARTD transform are assessed by calculating the mean absolute percentage transform fitting error ^FIT^ *ϵ* by

ϵFIT=∑n=1N|Y″−Ỹ″Y″|×100%l 22

**Figure 6:**
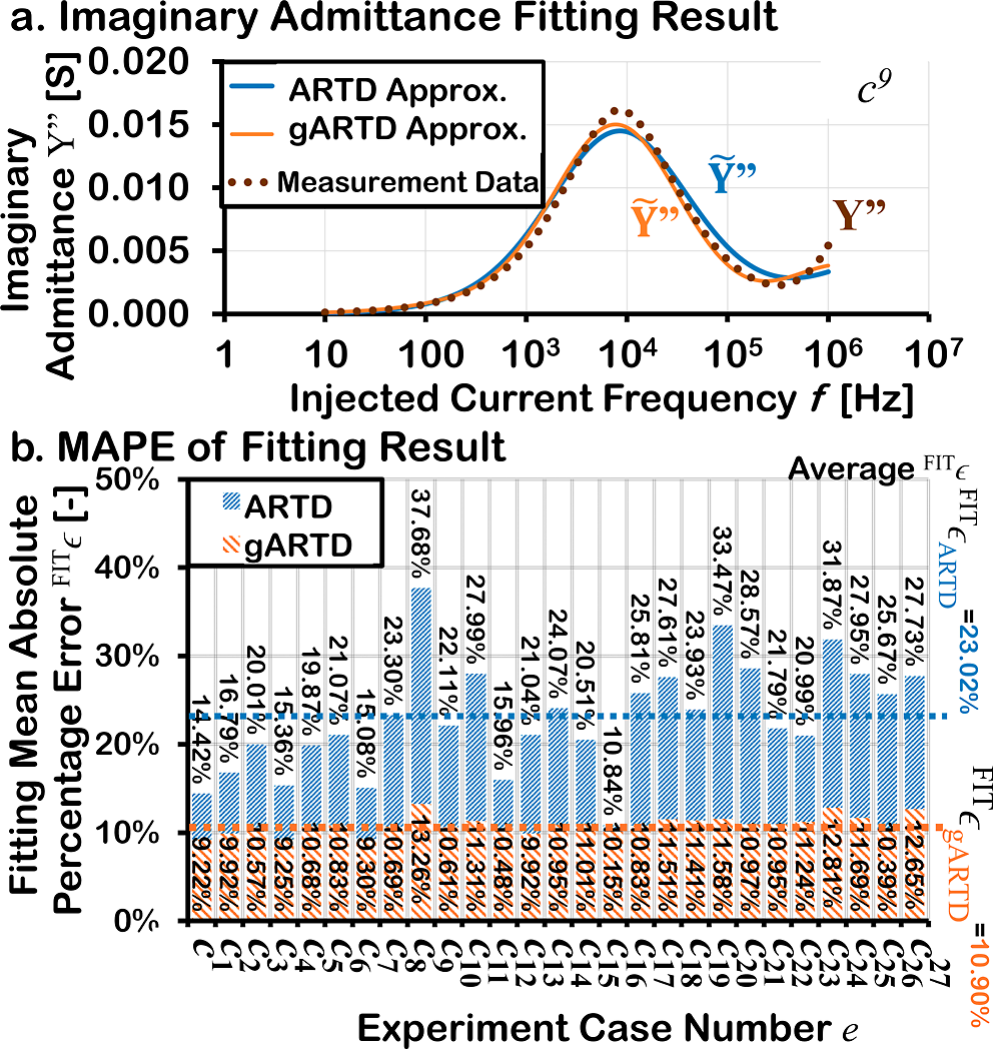
(a) Fitting results by gARTD and ARTD in Case *c* ^9^. (b) Fitting mean absolute percentage error ^FIT^ *ϵ* of gARTD and ARTD fitting in twenty-seven cases.

Figure 6b shows the ^FIT^ *ϵ* in gARTD and ARTD fitting, which guarantees the transform fitting error in twenty-seven training cases. The *ϵ* values of both methods in solution measurement are relatively small (< 40%), which is acceptable for fitting purposes. Based on the average of all the results in Figure 6b, gARTD has an average ^FIT^ *ϵ* of 10.90% and ARTD has an average ^FIT^ *ϵ* of 23.02%, which shows gARTD has a lower transform fitting error in comparison to ARTD. The higher transform fitting error occurring on ARTD is attributed on the reliance of ARTD on the **Y**″ distribution to follow the normal distribution along the relaxation times spectrum to minimize error, which does not always occur in actual measurements.

The prediction of peaks in gARTD and ARTD function provides a quantitative approach to analyzing the impact of protein and sodium electrolyte concentration. Figure 7 shows the gARTD and ARTD distribution functions of case *c* ^9^. Each method produces a peak at high relaxation times ( $\hat{\tau_{1}^{*}\;}$ > 0.5 ms) and a peak at low relaxation times (10 μs > $\hat{\tau_{2}^{*}\;}$ > 1 μs). The gARTD method produces an additional peak at $\hat{\tau_{1a}^{*}\;}$ ≈ 0.05 ms, where $\hat{\gamma_{1a}^{*}\;}$ is smaller than peaks $\hat{\gamma_{1}^{*}\;}$ and $\hat{\gamma_{2}^{*}\;}$ . The small $\hat{\gamma_{1a}^{*}\;}$ peak is eliminated by the peak points detection algorithm in eq 15 by declaring *γ*_lim_ = 4 mS. Figure 8 shows that the overall increase in protein concentration and electrolyte sodium concentration causes an increase in $\hat{\gamma_{1}^{*}\;}$ , which is affected by the counterion effect.^12,29^ The increase in $\hat{\gamma_{1}^{*}\;}$ is consistent with the free electric charge increase within the protein solution for the increase of all the target analytes concentration. Figure 9a shows that an increase in *c*_Alb_ causes an increase in $\hat{\gamma_{1}^{*}\;}$ shown in green arrows, and a decrease in $\hat{\tau_{1}^{*}\;}$ and $\hat{\gamma_{2}^{*}\;}$ is shown in red arrows. Figure 9b shows that an increase of _Glo_ *c* causes an increase in $\hat{\gamma_{1}^{*}\;}$ and $\hat{\gamma_{2}^{*}\;}$ and a decrease in $\hat{\tau_{1}^{*}\;}$ . Figure 9c shows that an increase in ^Na^ *c* causes an increase in $\hat{\gamma_{1}^{*}\;}$ and $\hat{\tau_{1}^{*}\;}$ . The increase in $\hat{\gamma_{1}^{*}\;}$ from ^Na^ *c* increase reaffirms the correlation between $\hat{\gamma_{1}^{*}\;}$ and the counterion effect, which is highly affected by the increased free electron provided by the sodium.

**Figure 7:**
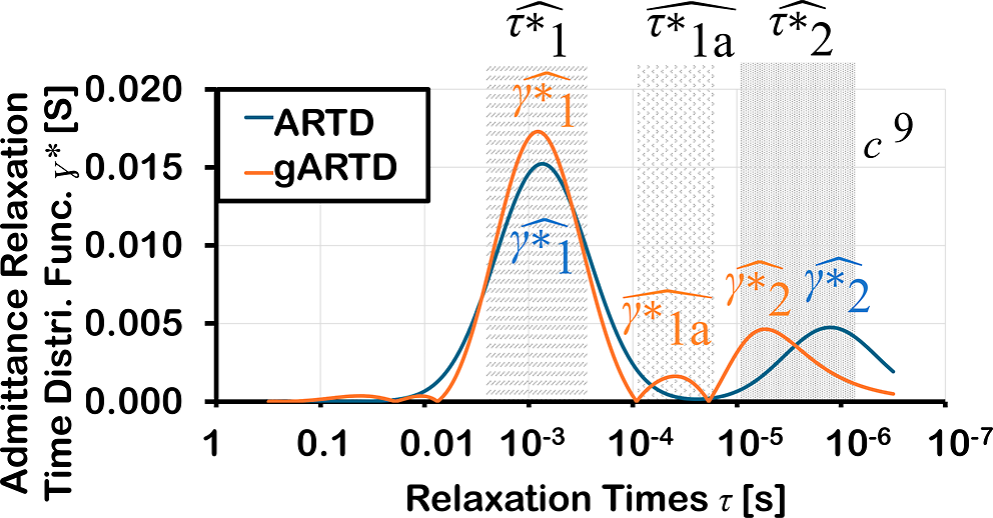
ARTD and gARTD distribution function in case *c* ^9^.

**Figure 8:**
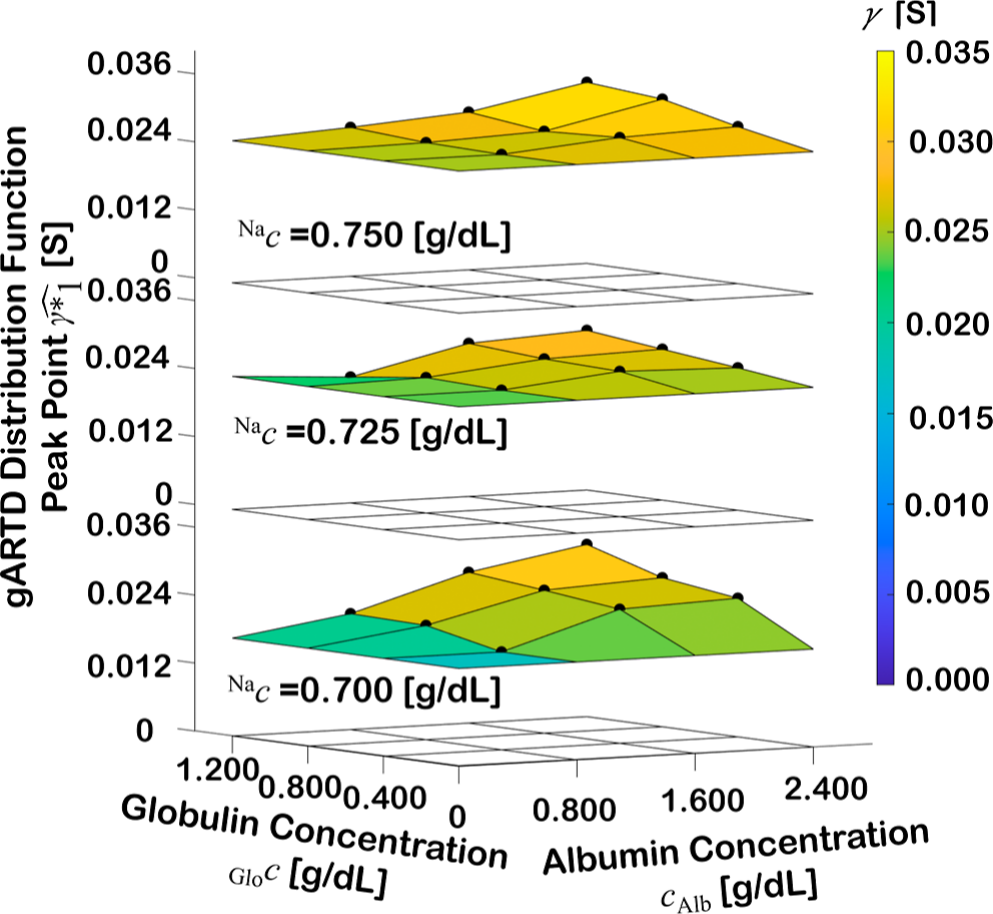
Impact of *c*_Alb_, _Glo_ *c*, and ^Na^ *c* increase on $\hat{\gamma_{1}^{*}\;}$ .

**Figure 9:**
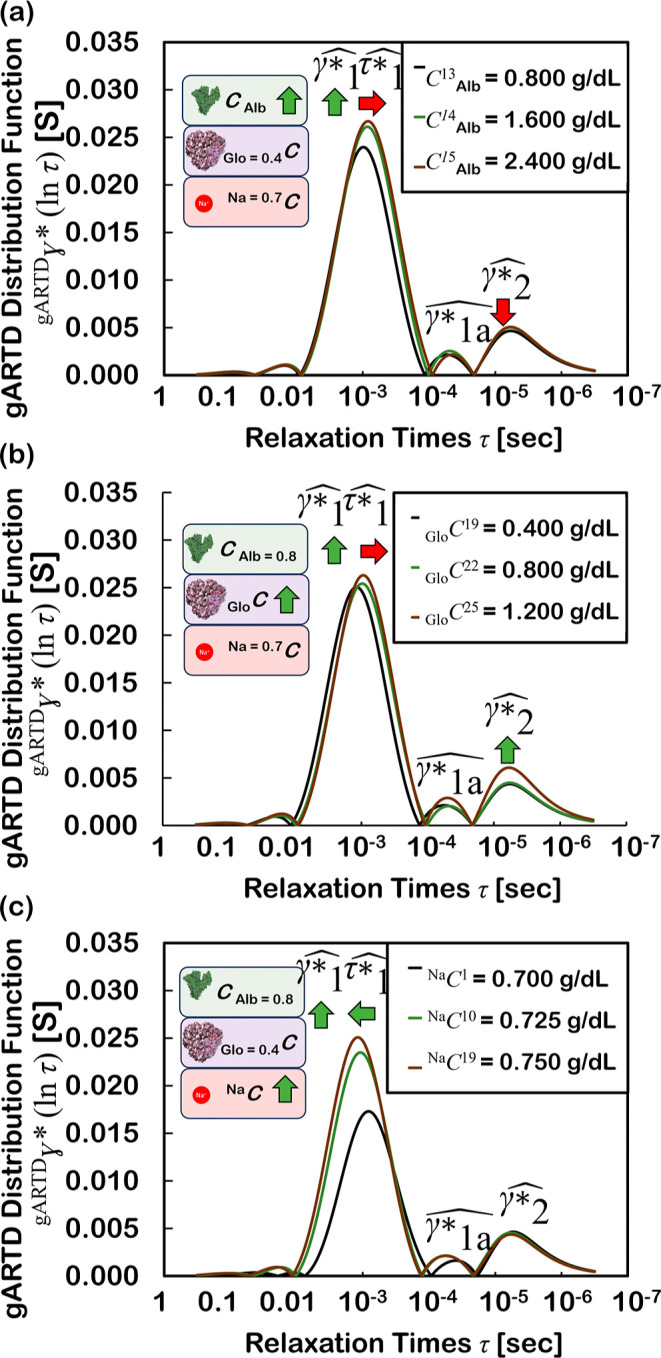
GARTD function of increasing substance concentration increase. (a) The gARTD function of increasing albumin concentration. (b) The gARTD function of increasing γ-globulin concentration. (c) The gARTD function of increasing sodium electrolyte concentration.

### 4.2 Calculation Result of Ã

The mgARTD approximated **Ã** is designed to quantify *c*_Alb_, _Glo_ *c*, and ^Na^ *c* from the gARTD transform of the twenty-seven experimental cases and admittance measurement **Y** ^+^. The gARTD function consists of two peak values (*Q* = 2), producing P* as the input from the training data set. The matrix **Ã** is calculated by eq 12 with P* and **c** as inputs. The approximated mgARTD coefficient ^mgARTD^ **Ã** ∈ R ^4×3^ is written as follows:

23 ÃmgARTD=[4.42×10−3−0.13×10−3−0.09×10−30.26×10−63.95×10−30.07×10−3−0.02×10−30.28×10−628.26×10−39.08×10−31.41×10−36.38×10−6]

while the approximated mARTD coefficient ^mARTD^ **Ã** ∈ R ^4×3^ is written as follows:

24 ÃmARTD=[4.34×10−30.05×10−3−0.08×10−3−0.12×10−64.15×10−30.34×10−30.01×10−30.15×10−620.69×10−34.81×10−31.24×10−37.07×10−6]

### 4.3 Quantification of Concentration by mgARTD and mARTD

The determination of **Ã** enabled quantification of *c*_Alb_, _Glo_ *c*, and ^Na^ *c* by mgARTD and mARTD. Figure 10 shows the accuracy evaluation of cases c^8^, c^11^, c^17^, c^18^, c^23^, and c^25^ by mgARTD and mARTD with the evaluated case concentration as *č*. The mgARTD produces quantification results with an average *ϵ* of 11.05% for *c*_Alb_, 11.55% for _Glo_ *c*, and 5.64% for ^Na^ *c*. The mARTD produces a higher *ϵ* on average, with an average *ϵ* of 21.20% for *c*_Alb_, 22.49% for _Glo_ *c*, and 13.38% for ^Na^ *c*. Both methods are able to quantify *c*_Alb_ and _Glo_ *c*, with ^Na^ *c* producing the lowest error. Figure 11 shows the quantification result of _Glo=0.400_^Na=0.700^ *c*_Alb=1.200_ for the protein solution. The mgARTD produces quantification results with an *ϵ* of 9.90% for *c*_Alb_, 9.12% for _Glo_ *c*, and 1.70% for ^Na^ *c*. The mARTD produces an *ϵ* of 25.56% for *c*_Alb_, 20.90% for _Glo_ *c*, and 25.71% for ^Na^ *c*. The mgARTD produces a smaller error on average than does mARTD.

**Figure 10:**
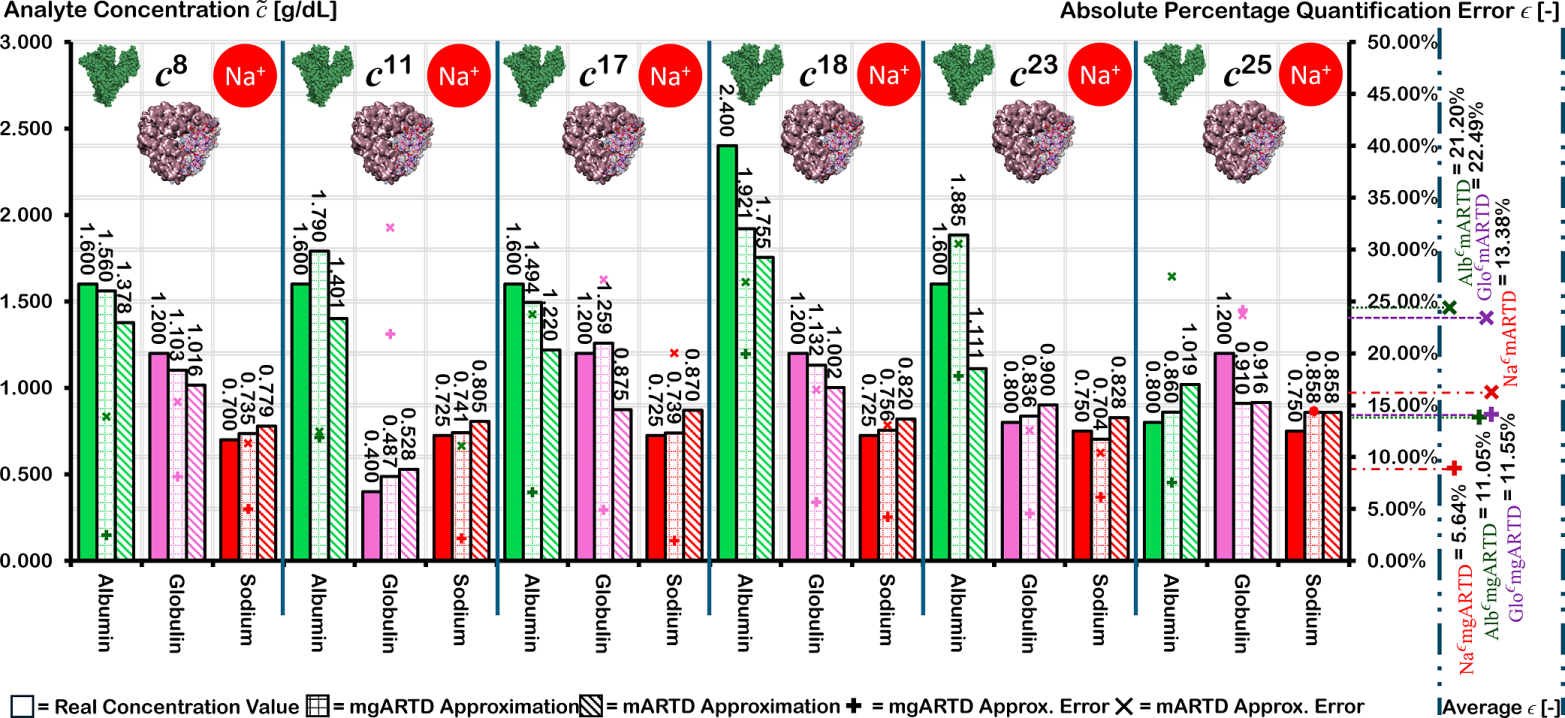
Evaluation of mgARTD and mARTD concentration for case c^8^, c^11^, c^17^, c^18^, c^23^, and c^25^ validation data with the average ϵ for each substance.

**Figure 11:**
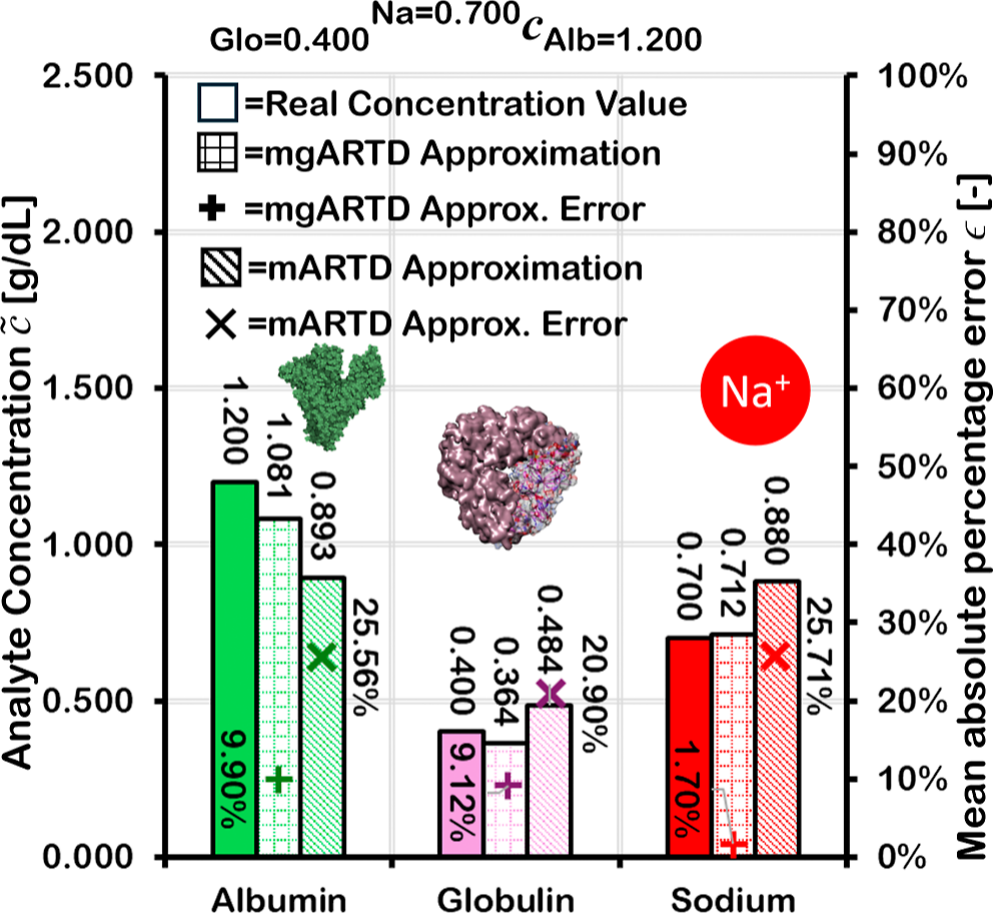
Evaluation of mgARTD and mARTD concentration quantification in validation data case _Glo=0.400_^Na=0.700^ *c*_Alb=1.200_.

## 5 Discussion

This study aims to assess the capability of mgARTD to quantify albumin and γ-globulin protein concentrations under electrolyte sodium fluctuation. The change in concentration impacts the dielectric relaxation times τ̂ and RTD function peak point γ̂. The $\hat{\gamma_{1}^{*}\;}$ in $\hat{\tau_{1}^{*}\;}$ > 0.5 ms is affected by the protein and sodium electric charge,^21^ and $\hat{\gamma_{2}^{*}\;}$ in 10 μs > $\hat{\tau_{2}^{*}\;}$ > 1 μs is primarily affected by protein β dispersion. The increase in *c*_Alb_ and _Glo_ *c* had an impact on $\hat{\gamma_{1}^{*}\;}$ , as shown in Figure 9a,b, due to the increased number of free electric charges within the solution and the formation of counterion. The phenomenon on $\hat{\gamma_{1}^{*}\;}$ also occurs in ^Na^ *c* increase, which reaffirms the impact of free electric charges^30^ on the mgARTD as shown in Figure 9c. The substantial impact of ^Na^ *c* on the additivity of protein solution also impacts the $\hat{\gamma_{2}^{*}\;}$ peak, as shown in Figure 9c, which is theoretically only affected by protein concentration due to the reorientation of protein.^31^ The application of gARTD reduces the average ^FIT^ *ϵ* by 12.12%, as shown in Figure 6b, which is a significant reduction in the transform fitting error. The improvement is attributed to the nonparametric approach of the Gaussian process with an infinite number of variables,^25^ which improves upon the ARTD. The $\hat{\gamma_{1a}^{*}\;}$ peak provided by mgARTD, shown in Figure 7, demonstrates the impact of an additional relaxation times phenomenon, which is outside of typical parameters for the counterion effect,^32^ protein reorientation, and protein β dispersion. The validation and testing of mgARTD demonstrate a significant improvement compared to mARTD. The quantification of protein by mgARTD in validation data produces an *ϵ* = 11.05% for *c*_Alb_ and *ϵ* = 11.55% for _Glo_ *c*, as shown in Figure 10, which is a substantial improvement upon mARTD. The testing result of mgARTD with measurement case _Glo=0.400_^Na=0.700^ *c*_Alb=1.200_ demonstrates an *ϵ* = 9.90% for *c*_Alb,_ *ϵ* = 9.12% for _Glo_ *c*, and 1.70% for ^Na^ *c*, which is significantly lower than *ϵ* = 25.56% for *c*_Alb_ and *ϵ* = 20.90% for _Glo_ *c*, and 25.71% for ^Na^ *c* by mARTD, as shown in Figure 11. Future studies require more cases, and smaller analyte concentration increments in the training data are required to optimize **Ã** further.^33^ The result of the experiment demonstrated the capability of mgARTD to quantify albumin and γ-globulin protein concentrations under electrolyte sodium fluctuation and improvement upon mARTD. A direct comparison of mgARTD with ELISA and NanoDrop devices under identical or comparable experimental conditions is currently unavailable, which makes a direct absolute percentage quantification error comparison inappropriate. Nevertheless, comparison of mgARTD results with the true concentrations of albumin, γ-globulin, and sodium electrolyte provides strong evidence of its capability for accurate multisubstance concentration quantification. Future studies are warranted to address this gap by performing side-by-side evaluations under identical experimental setups, thereby enabling a rigorous assessment of the practicality, cost-effectiveness, and accuracy of the proposed method relative to those of conventional techniques.

## 6 Conclusion

The multivariate-regression Gaussian ARTD (mgARTD) has been applied to quantify *c*_Alb_ and _Glo_ *c* under fluctuating ^Na^ *c*. The results are shown as follows:

1. The mgARTD transform demonstrates the impact of *c*_Alb_, _Glo_ *c*, and ^Na^ *c* changes on ARTD function $\hat{\gamma_{1}^{*}\;}$ in $\hat{\tau_{1}^{*}\;}$ > 0.05 ms for counterion effect and $\hat{\gamma_{2}^{*}\;}$ in 10 μs > $\hat{\tau_{2}^{*}\;}$ > 1 μs for protein reorientation.
2. The gARTD demonstrates an average transform fitting error of 10.90%, which is 12.12% lower in comparison to ARTD.
3. Quantification of protein solution demonstrates an absolute percentage quantification error *ϵ* of 9.90%, 9.12%, and 1.70% by mgARTD which is lower than *ϵ* of 25.56%, 20.90%, and 25.71% by mARTD for _Glo_ *c*, _Glo_ *c* and, ^Na^ *c*, respectively, in _Glo=0.400_^Na=0.700^ *c*_Alb=1.200_ testing data case.
